# Hydrogel Drug Delivery Systems for Bone Regeneration

**DOI:** 10.3390/pharmaceutics15051334

**Published:** 2023-04-25

**Authors:** Long Bai, Gang Tao, Maogeng Feng, Yuping Xie, Shuyu Cai, Shuanglin Peng, Jingang Xiao

**Affiliations:** 1 Department of Oral Implantology, The Affiliated Stomatological Hospital of Southwest Medical University, Luzhou 646000, China; 2 Department of Oral and Maxillofacial Surgery, The Affiliated Hospital of Southwest Medical University, Luzhou 646000, China; 3 Luzhou Key Laboratory of Oral & Maxillofacial Reconstruction and Regeneration, The Affiliated Stomatological Hospital of Southwest Medical University, Luzhou 646000, China

**Keywords:** hydrogels, drug delivery systems, bone regeneration, cartilage regeneration, mesenchymal stem cells, bone immunomodulation, tissue engineering

## Abstract

With the in-depth understanding of bone regeneration mechanisms and the development of bone tissue engineering, a variety of scaffold carrier materials with desirable physicochemical properties and biological functions have recently emerged in the field of bone regeneration. Hydrogels are being increasingly used in the field of bone regeneration and tissue engineering because of their biocompatibility, unique swelling properties, and relative ease of fabrication. Hydrogel drug delivery systems comprise cells, cytokines, an extracellular matrix, and small molecule nucleotides, which have different properties depending on their chemical or physical cross-linking. Additionally, hydrogels can be designed for different types of drug delivery for specific applications. In this paper, we summarize recent research in the field of bone regeneration using hydrogels as delivery carriers, detail the application of hydrogels in bone defect diseases and their mechanisms, and discuss future research directions of hydrogel drug delivery systems in bone tissue engineering.

## 1. Introduction

Large bone defect is a common clinical disease that can occur at all ages. Bone loss often leads to irregular shapes and incomplete organ functions, and can have adverse psychological and social effects on patients [1]. There are many causes of bone defects, such as trauma, inflammation and complications of surgery. However, the repair of large bone defects (>6 cm) still faces many difficulties. For example, the repair of long bone defects requires long treatment times, high costs and difficult surgery. Bone tissue engineering, including mesenchymal stem cells (MSCs) capable of differentiating into osteoblasts, factors that induce the growth and differentiation of osteoprogenitor cells and resorbable biological scaffolds [2,3,4,5], is considered a promising approach to address the challenges of bone defects. The major MSCs in bone tissue engineering are bone marrow mesenchymal stem cells (BMSCs), and the main osteogenic differentiation-inducing factors are bone morphogenetic proteins (BMPs) [6]. Bone scaffold materials commonly include β-tricalcium phosphate (β-TCP) [7] and 3D-printed biological scaffolds and hydrogels [8], which have been widely used in regeneration fields in recent years [9,10,11].

Trauma and various systemic diseases involving bone tissue, such as diabetes and congenital bone dysplasia, are the primary causes of bone defects [12,13]. Trauma-induced bone defects mostly occur in craniofacial bones and long bones of the extremities [14], and their incidence has been on the rise in recent years with the development of transportation [15]. Bone defects caused by various neoplastic and inflammatory diseases, such as maxillary and mandibular bone defects after maxillary tumor resection and bone defects caused by osteomyelitis-induced osteonecrosis, are also common [3,16]. Congenital bone development defects, such as a cleft palate, are often observed in the maxillofacial area [17]. Autologous bone grafting is undoubtedly the best way to repair small bone defects, with advantages of a low immune rejection rate, high survival rate, and easy access to materials [18]. However, for patients with severe systemic disease and large bone defects, autologous bone grafting is often limited [19]. As a result, allogeneic bone grafting has been developed to overcome the limited sources of autologous bone grafting. Allogeneic bone grafting can repair relatively large bone defects; however, there are cases of allogeneic bone rejection, resorption and even bone discontinuity [20]. On this basis, bone tissue engineering relying on synthetic materials has been developed with the help of stem cell engineering [21]. Implanting materials with similar structures and functions as bone tissue, such as polymethylmethacrylate (PMMA) and hydrogels [21,22,23], promotes angiogenesis and bone deposition in areas of bone defects [24].

In the past decade, research on nanotechnology-based drug delivery mechanisms and tissue engineering has made tremendous progress and has great potential to improve human health [25]. Nanomaterials have been shown to be carriers with different in vivo and vitro properties for targeted drug therapy to treat various diseases [26]. Hydrogels are a novel nano-drug delivery tool, with biocompatibility, biosafety, and excellent drug release control properties [27,28]. Traditional hydrogels have poor physical properties that limit their application, such as micron-sized hydrogel poly-N-isopropylacrylamide [29]. Nanotechnology-based composite hydrogels show better responsiveness and drug delivery capabilities, which are related to the type of reactive elements designed internally, such as responses to pH, temperature, pressure and light [30]. Nanoscale hydrogels are considered promising drug delivery vehicles in the medical field for controlling the rate and timing of drug release according to different stages of the disease [31]. Hydrogel-based drug delivery systems are increasingly applied in the field of tissue engineering [32], and are becoming an important material for bone regeneration and defect repair as an alternative to bone grafting. Hydrogels are gels with a hydrophilic three-dimensional mesh structure that has good water insolubility, allowing it to retain water [33]. The quantity of water retention determines the physical properties of the hydrogel. The more densely cross-linked the network structure, the less space is available, and less water is retained [34,35]. Based on these features, hydrogels can be designed as drug-carrying materials with certain strengths, hardness, and shapes [36]. They can be combined with 3D bioprinting ink materials to provide stability [37] and form a hydrogel drug-carrying system. Common hydrogel materials are natural hydrophilic polysaccharide polymers such as cellulose [38], sodium alginate [39], hyaluronic acid (HA), and chitosan [40]. Natural peptides, such as gelatin, and synthetic materials including PMMA and polyacrylamide, are also used for delivering drugs. The binding mode of the molecules inside the hydrogel is enriched, while the drug-carrying load of the internal molecules is enhanced by electrodialysis [41], microfluidics [42], physical cross-linking [43], and click reactions [44]. The characteristics of hydrogel drug release control require certain conditions for activation. The conventional hydrogel is not sensitive to temperature, pH, or other variables; however, by redesigning the molecules inside the hydrogel and changing their linking method, a drug release system can be formed that can sense features of the external environment such as the temperature, small changes, or a stimulus. For example, an increase in reactive oxygen species (ROS) content often suggests local oxidative stress and inflammation [45]. Researchers designed ROS-sensitive hydrogels based on the specific elevation of ROS at the site of bone defects [46,47]. Gels connect ROS scavenging catalytic nanoparticles through internal organic groups and trigger the release of ROS-scavenging nanoparticles when sensing an elevated external ROS concentration [48], reducing the amount of ROS in the bone defect area for anti-inflammatory purposes and promoting bone healing [49].

The main components of hydrogel drug delivery systems are nano- or micron-sized particle molecules. As an alternative treatment in the field of bone regeneration, hydrogel particles are designed in different diameters (1–1000 μm). Hydrogels can be designed as three physical states, hydrogel suspension, granular hydrogel, or hydrogel complex, depending on the experimental needs. In the field of bone regeneration, hydrogel suspensions and hydrogel complexes are widely used [50]. The production methods of hydrogels have also been improved by advancements in technology. Five methods are commonly used: microfluidic emulsion, electrohydrodynamic spraying, batch emulsion, lithography, and mechanical fragmentation. Batch emulsion is when the hydrogel precursor is dropped directly into the oil, then vortexed and mixed. The hydrogel particles are obtained by cross-linking and washing. The batch emulsion process is fast and easy; however, the resulting hydrogel particles are not uniform in size [51]. The mechanical fragmentation method is also relatively simple; it uses a fragmentation device to split the large hydrogel precursor into smaller hydrogel particles [52]. However, the final hydrogel particles are all large in diameter, with a minimum size of approximately 20–50 μm. The microfluidic emulsion approach is more generally recommended because it has low variability and a relatively small diameter. The hydrogel precursor and oil are delivered simultaneously in a fixed diameter pipe, with precise control over the amount of hydrogel each time. The hydrogel precursors are formed into spheres under the pipeline flow, and finally, after cross-linking and washing, hydrogel particles with a more uniform size and shape can be obtained. More importantly, the size of the channel can be changed to alter the diameter of the hydrogel particles. Additionally, the hydrogel produced by microfluidic emulsion is less toxic to cells than the other methods, and has a cell survival rate of >80%. Although this technology has been widely used, it requires that a low viscosity hydrogel solution and the formed droplets do not fuse immediately. Numerous experiments have shown that on-chip approaches are effective at improving the success rate of hydrogel fabrication with microfluidic emulsion [53].

The key feature of hydrogel complexes is the way in which the molecules within the hydrogel are bound. Chemical cross-linking is the main form of internal bonding, and it mainly depends on the basic components inside the hydrogel. For example, alginate is mainly bound to CaCl_2_ by ionic cross-linking, while methacrylate modified gelatin is cross-linked at elevated temperatures or UV irradiation [54]. Hyaluronic acid (HA) is a common class of hydrogel molecules that can be modified by a variety of chemical bonds. For instance, methacrylate, acrylate and tyramine are modified and react under UV light. Poly (ethylene glycol) (PEG) and poly (vinyl alcohol) (PVA) have rarely been used. PEG and PVA can bind a desired drug in the internal hydrogel molecules through the chemical cross-linking of vinyl sulfone and thiol and ene under UV irradiation [55]. The hydrogel complex is transported to the site of the bone defect and the hydrogel degrades to release drugs and cells for treatment.

This paper summarizes the applications of hydrogel drug delivery systems for bone regeneration in recent years, and hydrogels with MSCs for osteochondral defect repair are discussed, focusing on the mechanism of bone immunomodulation. Finally, the future direction of bone repair and the urgent issues that need to be addressed are outlined.

## 2. Hydrogel Drug Delivery Systems and the Repair of Bone Defects

### 2.1. Hydrogel Drug Delivery Systems and Cranial Defects

The repair of cranial defects is particularly important to isolate external stimuli and maintain normal brain function [56,57]. The repair of cranial defects is an important way to ensure the restoration of craniofacial function, and bone tissue engineering facilitates the repair of cranial defects by combining biocompatible materials, MSCs with osteogenic differentiation ability, and the functional extracellular matrix (ECM) [58,59]. Hydrogel drug delivery systems have great advantages in cranial defect repair by transporting the drug-resistant drugs quercetin and curcumin with hydrogels [60,61,62], which promotes osteoblast osteogenesis via the enzymatic response of the hydrogels to matrix metalloenzymes-2 to release drugs in response to enzymatic breakdown [63]. Hydrogels loaded with peptides by click chemistry or photopolymerization release the corresponding glycopeptides and integrin-binding peptide C into the defect [64,65,66,67]. By binding to macrophages and MSCs, the hydrogel forces the M1-type to M2-type polarization of the macrophages and the proliferation and migration of MSCs to achieve the anti-inflammatory promotion of bone regeneration [68]. Hydrogels can also be combined with macromolecules, and have shown excellent osteogenic and vascularizing abilities by combining 4-vinylbenzyl chloride. Phosphorylated functionalized chitosan and eggshell particles supported by 3D printed bio-scaffold polycaprolactone (PCL) expand the indications for hydrogel repair to various types of cranial defects [69,70,71,72]. Nanoscale hydroxyapatite enhances the stability and strength of the scaffold [69], allowing it to withstand certain external forces during the repair process [73]. Simple scaffold materials have been gradually replaced by composite scaffolds with growth factor inducers that can promote the osteogenic and angiogenic ability of MSCs by adding BMP-2 [23,74] and vascular endothelial growth factor (VEGF) [75,76,77]. Biological scaffold materials loaded with hydrogels and various types of osteogenic-vascular cells and cytokines have become the mainstream direction of bone defect repair (Table 1). Different components of hydrogels show different physicochemical properties, and biological materials also provide various functions for the scaffold, among which the types of MSC and their differentiation directions determine the bone defect repair [21]. Recently, mesenchymal ECM and extracellular vesicles have shown superiority as a novel stem cell therapy to promote bone regeneration, and they are expected to be alternative therapies for stem cell engineering in cranial bone repair [68,78,79,80].

### 2.2. Hydrogel Drug Delivery Systems and Jawbone Defects

Jawbone and alveolar bone defects are mainly caused by neoplastic diseases, inflammation, and trauma [87]. Bacterial and local microenvironmental alterations are important factors contributing to diabetic bone defects in corresponding maxillary and alveolar bone infections caused by the high content of glucose [88]. Antimicrobial and anti-infection characteristics are key to treating infected jaw defects. Xu et al. loaded hydrogels with bone enabling and photothermal antibacterial capabilities into 3D printed scaffolds and used them under near-infrared irradiation to remove Gram-negative and Gram-positive bacteria affecting osteogenesis in the area of a bone defect to promote bone regeneration [83,89]. The anti-inflammatory drugs resveratrol and interleukin-10 (IL-10) have significant advantages in promoting osteogenesis in infected bone defects when loaded in hydrogels by affecting the proliferation of MSCs, osteoclasts, and endothelial cells in the defect area and promoting the differentiation of macrophages to M2, secreting anti-inflammatory substances and exerting their ability to promote bone repair [84,90,91]. Dental pulp stem cells (DPSCs) are often used to repair alveolar bone defects because of their unique origin in the field of oral biotherapy [92]. Inflammation-induced alveolar bone loss is often also related to the state of the teeth, while the most common etiology in dentistry is periodontitis [93]. The combination of hydrogels with peptide and magnesium phosphate particles, wrapped around DPSCs, showed better biocompatibility and lower inflammatory properties than calcium phosphate alone under conditions without cell-inducing factor treatment [94]. Hydrogels are also compatible with gingival mesenchymal stem cell (GMSC) binding, promoting the expression of osteogenic factors such as RUNX2 in alveolar bone defects and eliminating peri-implant inflammation [95]. A composite hydrogel containing a novel functional peptide module, dithiothreitol, equipped with an antimicrobial short peptide, was prepared using a Michael addition reaction and released by injection into pathological periodontal pockets using specific cleavage in gingival proteases to clear the clearance of periodontal inflammation and promote proliferation, migration, and the osteogenic differentiation of periodontal ligament stem cells [96]. For the treatment of periodontitis and alveolar bone resorption, injectable hydrogels that can organically combine the removal of inflammation and the promotion of bone repair, and make the treatment process more comfortable for periodontitis patients, will be the future trend [97]. Physiological gingival crevices can store gingival crevicular fluid with antimicrobial capacity, and the development of periodontitis, can disrupt the normal gingival crevicular structure. Whether the injectable hydrogel can effectively protect the original physiological structure and maintain the body’s own immunity when treating periodontitis and other diseases still needs to be studied.

### 2.3. Hydrogel Drug Delivery Systems and Cartilage Defects

Articular cartilage is a special structure with complex functions that covers the surface of the bone for a cushioning function and reduces the friction during the movement of the joint. The purpose of cartilage tissue engineering is to remove inflammatory factors around the joints, promote chondrocyte regeneration and proliferation, and avoid joint degeneration and necrosis caused by the progression of arthritis, in order to restore the original physiological structure and function of cartilage [98]. Stem cells are still an important source for cartilage tissue engineering. Shen et al. subcutaneously implanted graphene oxide nanosheet hydrogels equipped with cartilage-inducing growth factor and BMSCs into the backs of mice, for which immunohistochemistry indicated cartilage-like tissue production after 4 weeks [99,100]. PFSSTKT (PFS), an affinity peptide sequence designed to promote the proliferation and differentiation of bone marrow mesenchymal cells, was combined with a hydrogel and a scaffold and placed in rabbit cartilage defects to recruit endogenous MSCs and promote cartilage tissue generation [65,101]. Infliximab has been shown to exert anti-inflammatory effects in the treatment of rheumatoid arthritis, mainly by reducing TNF-α expression to prevent further inflammation. The combination of an antibody and hydrogel provides a new repair idea by binding aldehyde groups to internal hyaluronic acid chains to reduce TNF-α upon local release in bone defects to promote bone defect repair [102]. Compared with direct antibody linking, the promotion of auto-local immunity could also effectively promote cartilage repair. The combined use of strontium and hydrogel cross-linked Bioglass released strontium to improve the secretion of glycosaminoglycans, the migration of hBMSCs, and the transformation of anti-inflammatory macrophage cells to M2 type by removing inflammatory mediators and promoting osteogenesis into cartilage [99,103]. Cartilage regeneration and repair focus on the removal of local inflammation [104], and increase the proliferation, migration, and differentiation of chondrogenic cells. Hydrogels enrich the pathway of cartilage defect repair with drug delivery and variable physicochemical properties, and they are expected to become an important vehicle for future cartilage regeneration and biomedical engineering fields.

### 2.4. Hydrogel Drug Delivery Systems and Osteochondral Defects

Osteochondral repair is a difficult area for bone regeneration because it is more difficult than simple bone regeneration and cartilage regeneration. Osteochondral defects are diseases that occur at the junction of bone and cartilage and are caused by bone and cartilage resorption because of inflammation and trauma [105]. Bone and cartilage are connected through the osteochondral interface, surrounded by muscle and fascia, forming a unique osteochondral functional body [106]. Bone and cartilage differ greatly because of their composition, physiological environment, and structure, and cartilage contains fewer chondrogenic cells and does not contain nutrient-rich blood vessels [107]. Therefore, bone tissue engineering still faces great challenges for repairing compound defects of osteochondral bone. Benefitting from the multidirectional differentiation potential of stem cells, stem cell engineering is still a hot topic for exploring the repair of osteochondral defects [108]. Hydrogels are an important bridge between bone tissue engineering and stem cell engineering because of their variable physicochemical properties, biocompatibility, simple fabrication, and accessibility for synthesis [109,110,111]. The semi-solid hydrogel carries drugs and MSCs and is injected locally into the joint cavity to promote the repair of osteochondral defects and the resolution of joint inflammation. Under near-infrared irradiation or enzymatic triggering, the hydrogel can locally release cells or protein molecules in response to the defect [110,112]. Hydrogels combined with a 3D-printed scaffold enhance chondrocyte migration and provide space for fibrocartilage attachment to protect new chondrocytes and promote the repair of bone defects [101].

**Figure 1:**
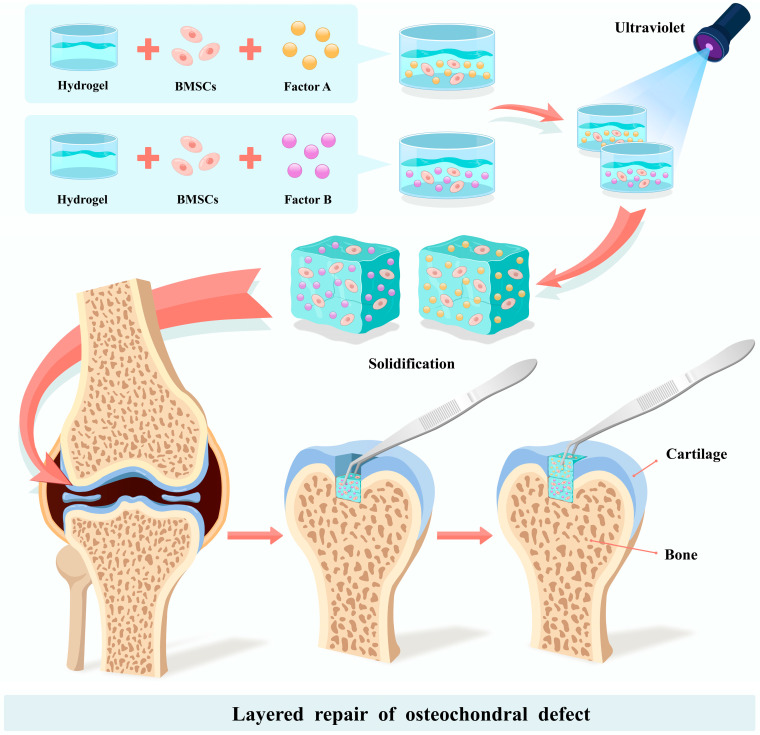
Liquid hydrogels and BMSCs were co-cultured in vitro with the addition of Factor A and Factor B, and the hydrogel mixture was cured with UV light irradiation. The cured hydrogel mixture was designed as a bone defect and cartilage defect, and it was used to fill the osteochondral defect area in layers. Factor A can promote the differentiation of BMSCs into chondrocytes and promote chondrogenesis. Factor B can induce the differentiation of BMSCs into osteoblasts and promote regeneration.

The traditional approach for repairing osteochondral defects is to implant a hydrogel with the flavonoid epimedium, which has osteogenic and chondrogenic properties, conjugated with hyaluronic acid (HA) and gelatin, and co-culture it with BMSCs. A fully layered osteochondral defect in a rabbit patella was treated with this method, and bone defect repair after one month and three months was demonstrated [40,113]. The shortcoming of this method is that it does not restore the physiological structure of the osteochondral contact interface. On this basis, Qiao et al. improved the efficiency of the osteochondral interface repair by cross-linking laminar fibers and growth factors inside the hydrogel to promote the regeneration of the fibrocartilage and bone tissue [114,115]. In response to the lack of cells and blood vessels in the fibrocartilage itself, articular cartilage progenitor cells that do not require special induction have also been used for osteochondral defect repair. The melt electro-writing (MEW) technique effectively combines hydrogel and bone bionic ceramic ink to carry articular cartilage progenitor cells, and has been shown to promote the deposition of the bone matrix and cartilage matrix in vitro [116]. For the osteochondral region with banded structural cartilage, the layered repair of bone and cartilage has become a new target. A bilayered hybrid hydrogel was prepared using thiol-ene cross-linkable HA and poly (glycidol), with the upper layer near the cartilage side carrying joint progenitor cells and the lower layer near the bone cortex carrying MSCs. The hybrid hydrogels were placed on osteochondral anchors and in knee defects of Shetland dwarf stallions, and six months later imaging and immunohistochemistry suggested that the osteochondral defects had corresponding bone and cartilage tissue production [117,118], suggesting that the layered repair of the osteochondral defect areas may be an important tool for the future. Biphasic hydrogels carrying BMSCs showed the ability to repair osteochondral defects compared with mixed hydrogels, where researchers cross-linked methacrylate-HA hydrogels with 2-isocyanatoethyl acrylate-modified β-cyclodextrin (β-CD-AOI2) and mixed them with MSCs in vitro [119]. A knee bone defect (2 mm deep) was injected with a mixed hydrogel containing the osteogenesis-inducing factor melatonin, and its coagulation was promoted by UV light during the injection [120]. In contrast, a mixed hydrogel containing the chondrogenesis-inducing factor kartogenin was injected into the upper cartilage defect (1 mm deep), and UV radiation promoted its coagulation to complete the biphasic repair of the osteochondral defect (Figure 1) [86]. The hydrogel delivery system offers bilayer repair of osteochondral defects, and it will become an important tool for cartilage regeneration and other osteochondral defects in the future [121,122].

## 3. Hydrogel Drug Delivery Systems and Bone Regeneration

### 3.1. Hydrogels and MSCs

MSCs are a class of stem cells with multidirectional differentiation and high proliferative capacity. They come from a wide range of sources and can be derived from bone marrow, fat, and dental pulp. Stem cells for clinical and engineering purposes fall into three groups: multipotent somatic stem cells, such as MSCs; pluripotent stem cells (PSCs), such as human embryonic stem cells (hESCs); and next generation stem cells. The third category is an edited stem cell based on potent chimeric antigen receptor (CAR) technology and the advent and evolution of CRISPR. Next-generation stem cells are considered the most promising engineered stem cells for treatment; however, technical and regulatory improvements are still needed [123]. Stem cells have two main roles in treating disease. The first is that they act as a vehicle for drugs. The second is the stem cells themselves are therapeutic drugs. In the treatment of bone defect repairs, MSCs can play a directly therapeutic role because of their excellent drug loading and facile combination with a hydrogel delivery system. MSCs in stem cell engineering include BMSCs and adipose derived stem cells (ADSCs), which can differentiate into various types of cells and form corresponding tissues to perform functions using their migration and proliferation ability [124,125,126]. BMSCs are the most commonly used MSCs in the field of bone tissue regeneration because of their homology with bone tissue [127].

Semi-solid gels can be specifically adapted to the size of the bone defect, and the internal space can support the morphology of the cells [128]. Additionally, the hydrogel can be injected for drug delivery, and it plays an important role in osteochondral repair [129]. In fact, although MSCs have multidirectional differentiation potential, they do not have the ability to differentiate into osteoblasts by themselves; they need be stimulated by various growth and differentiation factors to further transform into osteoblasts and chondrocytes. The success of the combination of hydrogel and bone marrow MSCs is largely because of the fact that hydrogel does not have toxic effects on the bone marrow MSCs and also has a cell-encapsulating effect [130]. Some scholars have applied MSCs and the ECM they secrete to the bone defect area and achieved an osteogenic effect. However, this method only achieved cell accumulation and had a weak effect on cell differentiation [131]. Hydrogels aggregate mesenchymal cells, and carry the factors that promote cell differentiation. More application scenarios for hydrogels are provided by 3D-printed biological scaffolds, and the scaffold material is modified several times to promote the migration and proliferation of the mesenchymal cells. The internal density of hydrogels can be designed to organize the cells into a mesh structure, which facilitates the directional transport of cells [132].

More recently, researchers have found that differences in the sex of the mouse affect the ability of bone stem cells to differentiate. Postmenopausal mice with osteoporosis showed a sharp drop in estrogen and had less osteogenic ability than the male mice at the same time. Studies have shown that mouse skeletal stem cell (mSSC) expression is positive for CD51 and CD200 and negative for CD105. When stimulated by growth differentiation factors such as estrogen, mSSCs can be CD105 positive and further differentiate into chondrocytes (CD200^+^ and Thy^+^), osteoblasts (Thy^+^), and stromal cells (6C3^+^). The activity of skeletal stem cells is a factor affecting bone formation, in addition to bone, cartilage, stromal progenitor (BCSP). BCSPs have a complex function and their activity is regulated by estrogen. Directly combining estrogen with poly (D, L-lactide-coglycolide) (PLGA) in the fracture area promotes osteogenesis. However, the PLGA fabrication process is complex and lacks stability; thus, an efficient and simple drug delivery system is needed to further improve drug delivery [133].

### 3.2. Hydrogels and Angiogenesis

Cell therapy has great potential in the field of tissue engineering and angiogenesis to promote bone regeneration [134,135]. Human umbilical vein endothelial cells (hUVECs) are important cells for vascular regeneration; however, one type of cell alone cannot restore all the functions of the regenerated tissue [135,136]. The mechanism by which hydrogels promote bone regeneration is mainly its ability to carry pro-angiogenic cells, which often creates a biphasic system with MSCs to accelerate the repair of bone defect areas. Hydrogel nanoparticles can cross-link various cellular particles, cytokines, and ECM, while carrying MSCs and UVECs, which are directed to the bone defect to mimic the normal biological environment around the bone to achieve bone regeneration and vascular regeneration [137]. In contrast to mixing UVECs and hydrogels and immediately implanting into bone defect sites, in vitro fibrin hydrogels co-cultured with UVECs provide a new method for tissue regeneration. Preformed, early vascular tissue is stable, and is more resistant to microenvironmental risk factors than those directly placed in the defect areas. The hydrogel is not toxic to endothelial cells, and the scaffold hydrogel acts as a “vascular cage” to protect the neovascular tissue [138]. The gap between the hydrogel and the scaffold determines the ability of BMSCs and UVECs to diffuse in the bone defect area [139], and the degradation rate of hydrogels determines the migration rate of cells, while the tissue-engineered reconstruction of bone and blood vessels requires them to be positioned as quickly as possible and aggregated at the defect site. The design of scaffold structures to promote the rapid formation of bone vascular tissue is considered a new direction. The laser femtosecond cautery technique processes the internal tubular wall of the scaffold with mesh-like pores, and hydrogel-loaded cells fill the pores with a cell diameter slightly smaller than the pore diameter, allowing for faster cell migration after the early implantation of the biomaterial [140].

### 3.3. Hydrogels and BMPs

BMPs are a class of transforming growth factors that induce the differentiation of mesenchymal cells into osteoblasts and chondrogenic cells [141,142,143]. For example, a stent material was equipped with BMP-2, which allowed BMP-2 to act directly on mesenchymal cells and promote their differentiation to osteoblasts [144,145]. BMP has a short half-life and is easily degraded in vivo, and hydrogels can be controlled to delay the degradation time. BMP is prone to inactivation and degradation when used directly. Binding to ECM can maintain its stability and reduce its degradation, thus prolonging the time to promote osteogenesis [146]. By increasing or decreasing the number and type of anions in the hydrogel composition and changing the solid–liquid transition period and internal pore size, a slow and sustained release of BMP-2 can be achieved to promote bone regeneration [147,148]. The early stage of drug release from hydrogels is explosive, and then the rate tends to level off [146]. It is difficult to use osteogenesis-related peptides, fibers, and polysaccharide macromolecules directly in clinical osteogenesis because of their unique physicochemical properties [75,130]. Fibrin hydrogels with large molecules of heparin, which are used to store BMP-2 and fibronectin to provide attachment to cells, avoid the burst release of BMP-2 at the beginning, prolonging the action of BMP-2 [147,149]. BMP-4 is also a transforming growth factor. A hybrid crystal system made of gelatin hydrogel cross-linked with heparin carried BMP-4 and VEGF to repair cranial defects, showing osteogenesis-related gene expression in alkaline phosphatase, alizarin red, and real-time PCR [76].

### 3.4. Hydrogels and Peptides

Peptides are a class of protein fragments with two or more amino acids condensed to form peptides with different chemical properties. The peptides can be designed to be antiviral, antibacterial, and able to mimic cytokines, such as transforming growth factors β (TGF-β), to promote myocardial, bone, and cartilage regeneration in vivo [150,151]. The mechanisms of peptides in repairing bone defects are mainly as follows. (1) Peptides act as TGF analogues directly on mesenchymal cells at the defect and promote their osteogenic differentiation. (2) Peptides act as vascular endothelial growth factor analogues to promote vascular regeneration. (3) Peptides promote the recruitment of stem cells at the defect. (4) Peptides improve the local microenvironment and promote the differentiation of macrophages to M2 type. The peptides act as a TGF to promote osteogenic differentiation; the responsive polymer acts as a “switch” when the osteogenic peptide is wrapped in a hydrogel and injected into the osteochondral defect of rabbit knee joint, resulting in the reactive release of osteogenic-related peptide GHK (GGGGHKSP) and chondrogenic-related peptide NC (GGGGHAVDI) that promote the osteogenic and chondrogenic differentiation of local MSCs and repair osteochondral defects [152]. VEGF is an important promoting factor for bone defect repair. Blood vessels provide the energy required for MSCs, and secreted growth factors promote the proliferation, growth, and differentiation of MSCs. QK (KLT-WQELYQLKYKG) is an important peptide that enhances VEGF binding and promotes vascular regeneration when paired with a hydrogel at 1:1 QK: RGD (GGGGRGDSP). The vascular growth factor, mineralization factor and mineralized nodules increase to boost local vascular infiltration and bone regeneration in rat cranial defects [153]. Stem cell homing technologies are considered to be a promising tissue engineering technique. Hydrogels and a scaffold material carrying a self-assembled peptide of functionalized bone marrow homing recruited endogenous stem cells and promoted their proliferation and differentiation into osteoblasts and chondrocytes, which has suggested better osteochondral repair via imaging and immunohistochemistry of the total cartilage defects of the rabbit knee joint after six months [154]. Glycopeptides mimic glycoproteins in the natural ECM and are usually assembled with fibers and gels to form artificial fibrous glycopeptide hydrogels (GH), which are combined with a biological scaffold to provide the corresponding strength and show significant osteogenic capacity in critical bone defects in rats. Glycopeptides promote bone regeneration, mainly for two reasons. On the one hand, they can mimic ECM, provide corresponding transforming factors, growth factors and signaling molecules, and they promote the proliferation and differentiation of BMSCs. On the other hand, they can polarize local macrophages of bone defects, transform them into M2 type with bone immunomodulatory function, secrete anti-inflammatory factors, and regulate each other with BMSCs to remove unfavorable factors, such as inflammatory factors, in the defect areas [57].

### 3.5. Hydrogels and Bone Immunomodulation

Bone immunomodulation has been one of the most important modalities in the field of bone regeneration in recent years. Aaron and Choi in 2000 first proposed bone immunomodulation in inflammatory bone diseases, explaining the interaction of the skeletal system with the immune system. The RANK signaling pathway is the main pathway through which osteoblasts associate with immune cells. Increased levels of RANKL induce the differentiation of the myeloid progenitor to osteoclasts. Within the bone tissue, osteoclasts and osteoblasts control the dynamic balance of bone mass [155].

#### 3.5.1. Hydrogels and Macrophage Cells

**Figure 2:**
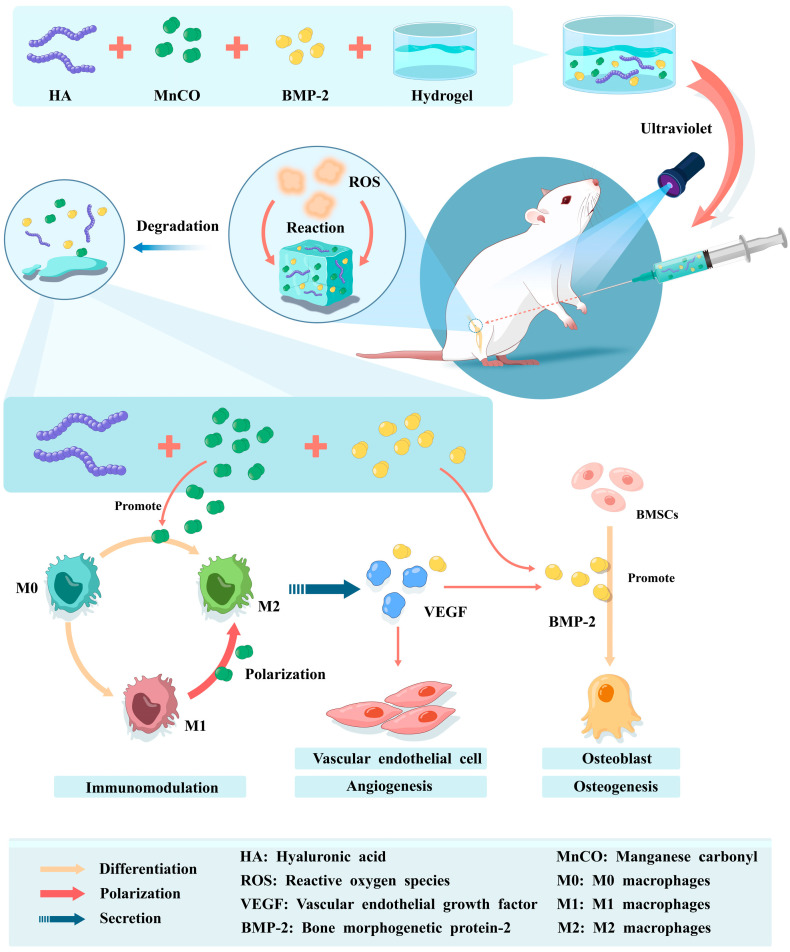
The hyaluronic acid hydrogel was cross-linked with MnCO and BMP-2, while the hydrogel was modified internally to be ROS sensitive. The hydrogel with the judgement-response feature was injected into the bone defect area of rats, and the hydrogel degraded after reacting with ROS, releasing the internal MnCO and BMP-2. MnCO can be degraded to Mn^2+^ and CO in the defect area, promoting macrophages to differentiate to M2 type and also causing M2 type macrophages to secrete anti-inflammatory factors VEGF and BMP-2, contributing to vascular regeneration and osteogenesis. The release of BMP-2 recruits local BMSCs, enhances their differentiation to osteoblasts and increases osteogenesis.

Immune cells and bone-associated cells constitute the main body of immunomodulation, which regulates the interaction between them and bone-associated cells by guiding, activating, stimulating, and inhibiting them [156]. At the same time, immunomodulation activates the immune system, improves the local microenvironment of bone defects, promotes osseointegration from a pathological state to a physiological state, and to some extent activates the host’s systemic immune defense system to carry out immune-related responses in concert [157,158]. Macrophages are one of the most important cells in the immune response, playing a regulatory role in the immune response [159,160]. M1-type macrophages produce inflammatory factors, causing a local inflammatory environment that disrupts the conduct of normal local physiological functions and damages the surrounding soft and hard tissues. When M1-type macrophage cells are affected by the microenvironment with an increase in surface antibody CD163, macrophage polarization occurs. Polarization is a dynamic process that reflects overall changes in macrophage function [161]. This process is stress feedback from the surrounding microenvironment to the organism and immune cells, explaining the important role of the immune microenvironment in the functional regulation of immune cells [162,163]. Manganese ions induce the early differentiation of macrophages to M2 type and prevent the production of pro-inflammatory factors (Figure 2). A methyl acrylate (MA) hydrogel combined with a PCL scaffold material and equipped with anti-inflammatory and antioxidant manganese carbonyl (MnCO), which reacts with hydrogen peroxide in the bone defect area in a Fenton-like reaction, releases Mn^2+^ and CO to promote macrophage M2 polarization and vascular endothelial factor release to repair the bone defect [37,164].

#### 3.5.2. Hydrogels and Osteoclasts

The stabilization of bone tissue is a dynamic balance between osteoblasts and osteoclasts. Osteoblasts secrete the bone matrix, which is mineralized and forms bone tissue. Osteoclasts are cells with bone resorbing activity which produce acids and proteases that digest and absorb the bone tissue that has been formed. Osteoblasts are mainly differentiated from MSCs in the bone marrow cavity and are mononuclear cells. Osteoblasts are polymorphic cells derived from the aggregation of myeloid progenitor cells or monocytes. Myeloid progenitor cells differentiate into osteoclasts and transform into macrophages via the macrophage colony stimulating factor (M-CSF) [165]. In bone defects in the inflammatory state, the levels of the inflammatory factors, such as interleukin-4 and interleukin-13, are dramatically increased. These inflammatory factors promote the conversion of mononuclear macrophages to multinuclear macrophages, and the fusion of multinuclear macrophages results in the formation of osteoclasts. In the treatment of inflammatory bone defects, hydrogels should promote the production and differentiation of osteoblasts, and inhibit the production of osteoclasts. An essential factor in this process is the receptor activator of nuclear factor κB ligand (RANKL), which has been shown to be a key factor in regulating the differentiation of myeloid progenitor cells to osteoclasts [166]. M-CSF induces myeloid progenitor cells to develop into macrophages. In contrast, RANKL and M-CSF acting together promote progenitor cells’ transformation into osteoblasts. Wei et al. noticed poor implant–bone bonding in patients with osteoporosis and designed a double-adhesive hydrogel. It has the effects of both promoting bone production by osteoblasts and inhibiting bone resorption by osteoclasts. The hydrogel is internally made with methacryloyl-HA, which allows it to collect with the calcium phosphate in the bone tissue. The hydrogel is modified by alendronate and bound to a bioactive glass modified by oxidized dextran. The stability of the implants in osteoporotic mice was greatly enhanced by both the hydrogel and Bioglass. In vitro experiments also revealed that the double-adhesive hydrogel upregulates the osteogenesis-related factor runt related transcription factor 2 (RUNX2) and downregulates RANKL; however, the exact mechanism needs to be further explored [167].

#### 3.5.3. Hydrogels and Osteoblasts

Hydroxypropylchitosan hydrogel combined with porous chitosan in an injectable drug delivery system on porous microspheres is the cornerstone of osteochondral defect repair. This new composite hydrogel promotes the proliferation and migration of BMSCs, while modulating the immune response and increasing M2 polarization. Dimethyloxyglycine (DMOG) plays a role in inhibiting the hypoxia-inducible factor (HIF) during hydrogel degradation and promotes macrophage polarization in a direction favorable to inflammation resolution and tissue repair [168]. M2 macrophages can also release TGF-β and VEGF to enhance the osteogenesis and vascularity of BMSCs, regulate the relationship between cells and the microenvironment, and can balance the relationship between promoting and inhibiting osteogenesis-related factors [169,170]. The hydrogel acts as a central control system, controlling the release of cells and drugs, controlling the adhesion of scaffold materials, directing stem cell migration, and releasing micro cytokines through its own response to the surrounding environment [171]. In inflammatory bone damage caused by diabetes, there is an excessive accumulation of local ROS, a deficiency of oxygen and an increase in nitric oxide (NO), often accompanied by infections such as oxygen-resistant *Staphylococcus aureus*. Hydrogel combined with pravastatin sodium and manganese dioxide (MnO) is injected locally to promote MnO decomposition by reacting to ROS to release a drug [172]. Mn^2+^ is an important regulatory factor that can play a role in immune regulation and anti-inflammation. The Mn^2+^ recruits macrophages to the defect and induces them to polarize toward the M2 type, while also removing excess ROS and reducing the damage to tissues and cells in the defect area from oxidative stress. Mn^2+^ also interferes with the bacterial metabolism and inhibits the proliferation of Gram-positive and Gram-negative bacteria, reducing the risk of tissue infection [173,174,175,176]. Skeletal immunomodulation has received increasing attention in recent years, and the focus of bone tissue engineering has gradually shifted to the regulation of the bone tissue microenvironment. Bone immunomodulation coordinates the state and function of cells and stabilizes normal immunomodulatory factors and growth factors. This is reflected in the use of existing materials to restore both hard and soft tissue in the defect area. The loading of immunomodulatory substances in hydrogels is an interesting attempt to restore the original biological environment of the defect area and to regulate the relationship between neutrophils, macrophages and osteoclasts in order to achieve the best repair effect [177].

### 3.6. Hydrogels and the Extracellular Matrix

The ECM is a network structure comprising extracellular macromolecules including proteins, proteoglycans, and polysaccharides [5,178]. The ECM is a mixture of collagen proteins that play a role in supporting and stabilizing the morphology and position of cells. In bone tissue, the ECM can be derived from osteoblasts and chondrogenic cells and is assembled and sheared extracellularly [179,180]. In recent years, researchers have observed that the ECM influences cell polarity by controlling extracellular signaling molecules and connects cells to form tissues and organs. The ECM can also stimulate cell migration and proliferation to regulate cell function. Hydrogels are considered an “artificial extracellular matrix”, mainly because of their natural internal components, such as chitosan and cellulose, which are similar to extracellular components. Hydrogels can restore the physiological environment of cells and also provide the corresponding transforming growth factors [181,182]. Hydrogels are considered to have promising applications in stem cell therapy because of their ability to mimic the action of the ECM. Combined with the liquid physical properties of hydrogels, they can be designed as injectable types that greatly enrich drug delivery routes, especially for closed cavities or surface bone defects. When tetra-arm poly PEGs and potassium sulfate cross-linked hydrogels are used for bone tissue repair, it can form ECMs with high toughness in situ and carrying chondrocytes, which can achieve better osteochondral defect repair [182,183]. Extracellular vesicles are a special class of ECM comprising lipid bilayer membrane structures, a class of tiny vesicles secreted by cells [184]. Extracellular vesicles carry small nucleotides, such as signaling proteins, miRNAs, and lncRNAs, that bind to target cell surface ligands and release intracellular substances that play a role in signaling and regulation [185].

### 3.7. Hydrogels and Exosomes

BMSCs’ exosomes are extracellular vesicles that have multiple functions and are often used as carriers to deliver nucleic acid molecules and signaling proteins in the field of tissue engineering. The exosomes secreted by the cells contain mainly small molecules of RNA, and the types of RNA encapsulated in the exosomes produced by BMSCs in various states are different. Compared with the exosomes from normal BMSCs, the exosome from hypoxia-treated BMSCs had a significant promotional effect on the growth of articular chondrocytes. This may be related to miR-205-5p in the exosomes of hypoxia-treated BMSCs, which promote cartilage defect repair through activation of the PENT/AKT signaling pathway. Injectable cellulose hydrogels encapsulate exosomes, protecting them from physicochemical stimuli while also slowing their release through their own degradation for continuous drug delivery [186]. The local microenvironment of diabetic bone defects is characterized by high concentrations of local ROS, inflammatory factors and blood glucose. A PEG/DNA hybrid hydrogel wrapped around the apical papilla secretes exosomes, relying on the responsive degradation of the hydrogel to matrix metalloproteinases in the surrounding environment, and slowly releases miR-150-5p, which promotes the expression of angiogenic and osteogenic-related genes through miR-126-5p cross-linking in the nucleus to repair bone defects [82]. Hydrogel-loaded exosomes for repairing bone defects are more workable and stable than hydrogels alone, which mimic the ECM and skeleton-loaded stem cells. Specifically, the hydrogel itself is responsive to degradation and cannot provide a relatively constant osmotic pressure on stem cells, which may lead to stem cell crumbling or rupture. Additionally, the hydrogel cannot simulate the normal cellular surroundings and cannot provide sufficient nutrients sustainably, and the stem cells wrapped for a long time may die because of the lack of nutrients. Exosomes, as extracellular vesicles secreted by cells, can tolerate nutrient deficiency and carry associated small molecule RNAs inside the vesicles to avoid degradation, which becomes an excellent signaling tool and is expected to play a greater role in the tissue engineering field.

### 3.8. Hydrogels and Nucleic Acid Molecules

The DNA molecule benefits from its precise base complementary pairing principle and hydrophilicity [187,188], which makes it highly adaptable to hydrogels. Depending on the carrier function, a hydrogel can carry the DNA and RNA signal molecules that contribute to bone, acting on the bone defect area to promote bone regeneration [189,190]. Hydrogels carry large molecules of natural and synthetic substances that may damage the normal tissues in the defect area, while DNA bioactive molecules will minimize the damage to the tissues and be more biocompatible. By cross-linking interleukin-10 with a ssDNA hydrogel and taking advantage of the slow-release effect of hydrogel and DNA to continuously act on an alveolar bone defect caused by diabetes, the hydrogel can effectively exert its anti-inflammatory and bone-building features [91]. DNA molecules can also form hydrogen bonds through their own complementary pairing, enhancing the linkage with the hydrogel molecules. The double-stranded DNA molecules denatured by the treatment are equipped with two-dimensional silicate nanodisks (nSi) that become active centers during gel solidification and increase the physical properties and mechanical strength of the hydrogel itself. DNA hydrogel carried the osteogenic drug dexamethasone and adipose stem cells (ASCs), such that the hydrogel carrier had an anti-inflammatory effect and osteoblast differentiation, and the repair of rat cranial defects in vitro showed a good bone regeneration performance [191].

The nucleic acid molecules contained in exosomes are mainly small messenger RNAs with more limited sources and a smaller variety, and the use of larger DNA or RNA molecules in exosomes is often restricted. With the intensive study of RNA molecules in signal transduction and transcriptional regulation, drug delivery systems using biological scaffolds or materials as carriers to carry RNA families for the treatment of bone defect diseases show great potential [192]. Single-stranded RNA molecules are highly susceptible to degradation; however, virus carrying RNA avoids degradation, while practical application and biosafety still pose great risks [193]. siRNA in gene therapy is mainly used to treat diseases by silencing relevant mRNAs, blocking their protein expression and inhibiting the level of post-transcriptional translational modifications of target genes [194]. Hydrogel-loaded siRNA is a major breakthrough in gene therapy that can effectively improve the speed and duration of siRNA transport while reducing the degradation rate of siRNA. Generation 5 poly amidoamine (G5-GBA) microspheres equipped with siRNA microsphere particles block the expression of the (TGF-β1)/Smad3 signaling pathway, avoiding periosteal fiber proliferation and adhesion and reducing the physical irritation of the hydrogel [195]. miRNAs can play a role similar to that of mRNA in promoting the expression of osteogenic and chondrogenic proteins, in contrast to siRNA. The conventional method is to transfect miRNAs into bone marrow MSCs in vitro, and then transfer the stem cells to the bone defect area for repair by the scaffold material. Although transfection is a more efficient method, the subsequent encapsulation and transformation steps of in vitro transfection are cumbersome. The hydrogel provides an environment that allows the in situ transfection of miRNAs within the hydrogel, which greatly diminishes the miRNA transfection time and also makes transfection efficiency more stable [196]. Nucleic acid molecules are highly degradable, and have a short duration of action when acting directly on the bone defect area. The molecules inside the hydrogel cross-link the nucleic acid molecules, which can maintain the stability of the nucleic acid and provide protection. The diversity of base complementary pairings offers more possibilities for the design of hydrogel molecules. By linking resiquimod (R848) and ovalbumin encoding mRNAs (mVOA) into the hydrogel through π–π stacking via electrostatic interactions, the continuous immunological effect of mRNAs can be ensured [197].

### 3.9. Hydrogels and the Microenvironment of a Bone Defect Area

The influence of the biological microenvironment on a hydrogel in the bone defect area is multifaceted. On the one hand, the specific components of the microenvironment activate the release or function of the hydrogel, which is mostly observed in the responsive hydrogel system carrying monomeric molecules. For example, in bone defects caused by chronic periodontitis, the ROS content in the local microenvironment is greater than that of the normal group, and through the activation of the hydrogel responsive release of doxycycline and metformin, local antibacterial and hypoglycemic effects are achieved and the surrounding inflammatory microenvironment is improved, thus promoting bone repair [48,198]. The design of responsive hydrogels requires two layers of logic, namely “judgment” and “response”, which modify their state by sensing the microenvironment and processing accordingly [199]. While a simple bone defect requires only a simply designed hydrogel, there are numerous dependent variables in the microenvironment of a bone defect site caused by a systemic disease. In diabetic bone defects, local negative factors include glucose fluctuation, ROS accumulation and a high expression of MMP, and the hydrogel designed for this microenvironment cannot focus on one aspect only [200,201]. The reversible cross-linking of PVA and gelatin designed as a multi-logistic hydrogel drug delivery system provides a new outlook in which high levels of glucose and ROS in diabetic bone defects can induce the degradation of PVA, while MMP acting on gelatin can reactively evoke the release of interleukin-10 and BMP-2 within the gelatin. In early repair, interleukin-10 promotes anti-inflammation, forces macrophages to polarize toward M2, produces anti-inflammatory factors, and improves the local inflammatory microenvironment, supporting BMP-2 to contribute to mesenchymal cell aggregation and differentiation into osteoblasts in late repair [164]. Alternatively, hydrogels act as part of the microenvironment involved in the function. When a hydrogel picks up cells, it acts like an ECM, protecting and supporting them. Different components of the hydrogel degrade to perform different functions; however, all hydrogels can envelope cells and deliver drugs and cells. Hydrogels can be formed from natural polymers and synthetic molecular polymerization, and their properties are determined by the internal molecules. Additionally, hydrogels can be used as a vehicle for carrying inorganic substances. Hydrogels can accommodate calcium ions that have the ability to promote osteogenic mineralization, and nanosheets that promote calcium ion trapping [202]. However, simple hydrogels as filler materials are gradually being substituted by composite hydrogels because of their lower mechanical strength and lower homogeneous composition. The temperature and pH of the microenvironment can change the physical properties of the hydrogel, while delivering the corresponding drug and cell [203]. Injectable hydrogels are widely applied because of their facile delivery, and the loading MSCs for bone regeneration is a typical method for bone tissue engineering. In order to create multiple forms of hydrogels, injection with simultaneous UV irradiation to promote solidification has become an essential step. With a photothermal responsive hydrogel, the hydrogel can fill the bone defect as much as possible while sustaining a certain mechanical strength [204].

## 4. Conclusions and Future Prospects

Bone tissue engineering materials, with advantages of low immunogenicity and biocompatibility, have introduced new hope for the repair of large area bone defects. The 3D-printed biomaterials are important vehicles for bone tissue engineering; however, improvements are still needed in terms of applicability and personalization. For example, 3D-printed scaffold materials are very stiff, and some defects require soft tissue. High strength scaffolds often cause impaired local soft tissue functional recovery. Therefore, a material with controllable morphology and adjustable hardness is needed. Hydrogels are transparent gels with a three-dimensional internal structure that can store water and have a certain toughness. Their physical properties are reversible under certain conditions, which gives them a wide range of applications [205]. Hydrogels show their unique prospective uses in the fields of tissue engineering and bone regeneration, with greater potential than purely biological scaffold materials. Hydrogels with MSCs are injected directly into the bone defect and responsively cured by photothermal treatment to support the cellular morphology and structure of the bone defect area. The process is simpler than 3D-printed biomaterials because it reduces the scaffold implantation time and provides a high level of biosafety [206,207].

In recent years, 3D-printed hydrogels have gradually become used for bone and cartilage repair [208]. This 3D printing technology aims to form biomaterials with homogeneous structure and stable strength using editable inks; however, unstable ink materials and unreasonable printing parameters severely hinder the formation of the final product [209]. The 3D-printed polyethylene glycol-diacrylate (PEDGA) and SA hydrogels have excellent strength and biosafety and can be cured rapidly using UV light, improving the stability of the printed material in all dimensions [210]. Meanwhile, 3D-printed hydrogels are more editable than traditional hydrogels, allowing unique rheological behaviors to be designed for bioinks and improving the mechanical properties of hydrogels, which are considered to have great potential for applications in responsive sensors and wearable sensors. However, the cumbersome printing procedure, the uncontrollable heat generation during printing, and the destructive nature of the ink outlet on the internal structure of the hydrogel remain major challenges for 3D printing hydrogels [211]. Therefore, conventional hydrogels still have certain scenarios of applicability.

**Figure 3:**
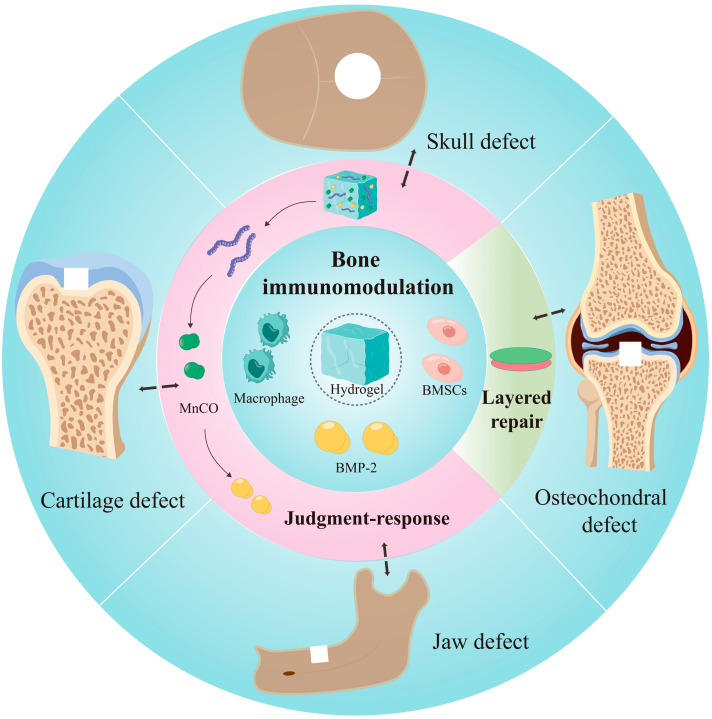
Relationships of hydrogel drug delivery systems and bone regeneration.

Repairing osteochondral defects has long been a major challenge in the field of bone regeneration. Trauma, osteoarthritis and infection can cause damage to bone and cartilage tissues. Because of their unique physical and biological properties, hydrogels are increasingly showing their advantages in the repair of osteochondral defects. Hydrogel drug delivery systems deliver cells, proteins, and nucleic acids into the body, facilitating the release and transformation of drugs. For the repair of osteochondral defects, layered design and biphasic repair are highlights of hydrogels, showing higher healing ability and functional recovery of the defect. Hydrogels promote bone regeneration and cartilage regeneration, restore the osteochondral interface, and reconstruct the biological function of both. Here, we summarized and proposed the concept of the “layered repair” of osteochondral defects, with the aim of providing a new way of thinking in the field of bone regeneration for repairing bone defects. We also clarified the important role of hydrogels in layered repair, in addition to their roles as cell carriers and drug carriers (Figure 3). Regarding the molecular mechanisms, the local microenvironment of bone defects can influence the response of hydrogels. Drugs can regulate the immune factors in the environment and promote the production of anti-inflammatory factors and the polarization of M2-type macrophages. Meanwhile, they restore the normal biological environment of the defect area to further promote bone regeneration. This paper summarized the application of hydrogels as drug delivery systems in the field of bone regeneration in recent years, especially in the treatment of large bone defects. We introduced the progress of research on the therapy of common bone defect diseases such as craniomaxillofacial bone defects, cartilage defects and osteochondral defects. We generalized the mechanism and principle of hydrogel delivery systems to promote bone regeneration. This article concluded with the layered repair protocol of hydrogels in osteochondral composite defects, and the specific mechanisms of their involvement in bone immunomodulation. In the future, hydrogel drug delivery systems in the field of bone regeneration will have great potential. Hydrogels with a layered design or multilayer logic design will become more and more comprehensive and responsive. Hydrogels with a “judgment-response” mechanism will be applied extensively, and they are expected to become a bridge between stem cell therapy and bone regeneration, thus helping to improve clinical treatment.

In conclusion, hydrogels have great potential for application in the field of bone regeneration because of their simple synthesis, high drug loading rate, low biosafety, and certain physical strength. However, the healing of bone tissue is not only dependent on external drugs alone, but also on the synergistic effect of various cells and tissues in the areas of bone defects. Bone regeneration still requires drugs that can both promote osteogenesis and coordinate the physiological function of the bone defect area, and even the immunity of the whole body. Hydrogels have been proven to be drug carriers in promoting local bone regeneration. In the future, it remains a great challenge to determine whether hydrogels can replace tissue-engineered scaffold materials and coordinate the dynamic balance of bone volume.

## Figures and Tables

**Table 1:** Hydrogel drug delivery systems used in various bone defects.

Types	Polymers/Monomers	Drugs	Methods	Features/Mechanics	Size	References
Cranial defects	Chitosan	Montmorillonite Riboflavin	Electrostatic interaction	Recruit native cells for bone regeneration	3 mm	[63]
	Glycosyl-nucleosyl-fluorinated (GNF) amphiphile	BMP-2	Incubated at 37 °C for 30 min	Slowly release BMP-2	3.5 mm	[81]
Jawbone defects	Polyethylene glycol (PEG)	DNA single strand, MMP-9 DNA aptamer-linker (Apt-linker), DNA hybridization	Thiol-ene click	Antibacterial, induces osteogenesis	4 × 2 × 1 mm	[82]
	Gelatin methacrylate (GelMA)	Sodium alginate (Sr^2+^), β-TCP, MXene (Ti_3_C_2_)	Mix in dark at 50 °C for 2 h, photo-crosslinked	Antibacterial, induces MSCs differentiation	5 mm	[83]
	Polycaprolactone/β-tricalcium phosphate (PCL/TCP) mixture	Resveratrol (RSV), strontium ranelate (SrRn)	Ultraviolet irradiation	Promotes MSCs proliferation and osteogenic differentiation	4 mm	[84]
Cartilage defects	Sodium alginate (SA), 1-ethyl-3-(3-dimethylaminopropyl) carbodiimide	Exosome	Chemical reaction	Recruits BMSCs and induces cartilage regeneration	5 × 3 mm	[85]
Osteochondral defects	Photocrosslinkable HA methacryloyl	Kartogenin, melatonin	Host–guest interactions	Induces cartilage regeneration and bone regeneration	6 × 3 mm	[86]

## Data Availability

Not applicable.
